# Developmental Trajectory of Depressive Symptoms from Early Childhood through High School in Children and Adolescents with a High Intellectual Potential

**DOI:** 10.3390/children10111738

**Published:** 2023-10-26

**Authors:** Laurence Vaivre-Douret, Soukaina Hamdioui

**Affiliations:** 1 Department of Medicine Paris Descartes, Faculty of Health, Université Paris Cité, 75006 Paris, France; 2 Clinical Neurodevelopmental Phenotyping, University Institute of France (Institut Universitaire de France, IUF), 75005 Paris, France; 3 Unit 1018-CESP, PsyDev/NDTA Team, National Institute of Health and Medical Research (INSERM), Faculty of Medicine, University of Paris-Saclay, UVSQ, 91190 Villejuif, France; soukaina.hamdioui@inserm.fr; 4 Department of Child Psychiatry, AP-HP Centre, Necker-Enfants Malades University Hospital, 75015 Paris, France; 5 Department of Endocrinology, IMAGINE Institute, Necker-Enfants Malades University Hospital, 75015 Paris, France; 6 Necker-Enfants Malades University Hospital, “Neuro-Développement et Troubles des Apprentissages (NDTA)”, INSERM UMR 1018-CESP, Carré Necker Porte N4, 149, Rue de Sèvres, 75015 Paris, France

**Keywords:** high intellectual potential, gifted children, adolescent, depression, developmental trajectory, psychomotor development, socio-affective relationships, social adjustment, learning disabilities, self-esteem, risk factors

## Abstract

We aimed to explore the developmental trajectory of depressive symptoms in a national sample of French children with a high intellectual potential (HIP) seeking help from gifted organizations. Participants were drawn from a national retrospective survey sent to 1200 families with HIP children (IQ ≥ 130) from primary to high school and they answered a self-report questionnaire of a depression scale (MDI-C). The children’s parents completed a self-report questionnaire collected on different stages of the child’s school level, perinatality, psychomotor development, health, family’s history, behavior, interpersonal relationships and daily activities, school performance, presence of learning disorders and remediation. Four hundred and twenty HIP children were eligible with an IQ ≥ 130 aged from 8 to 17 years-old, 49% with depressive symptoms and 51% with no depressive symptoms. Analysis of 136 variables from anamnestic fields based on the use of Spearman’s ρ test (ρ) with a non-parametric correlations showed that “learning disabilities” are significantly related to depressive symptoms in different groups (primary *p* = 0.001, middle *p* = 0.02, high school *p* = 0.001) as well as “difficulties in psychomotor skills” during primary (*p* = 0.003) and middle school (*p* = 0.02). Good relationships with family as well as with peers are significantly negatively correlated with depressive symptoms from childhood to primary (*p* = 0.003) and high school (*p* = 0.02). Certain details of correlations between the MDI-C scale’s subfactors and anamnestic variables were analyzed. The ANOVA test about the MDI-C scale showed provocation as a significant marker at middle school (F (1, 418) = 3.487, *p* = 0.03) and low self-esteem at high school (F (1, 418) = 3.337, *p* = 0.03). A holistic developmental approach allowed us to highlight the risk factors of depression with a developmental trajectory origin linked to disorders of social adjustment and psychomotor skills and to the importance of misdiagnosed learning disabilities because of giftedness. Our findings support the interest in an early identification of and intervention in depression risk to improve clinical decision making on the effect of giftedness on mental health outcomes.

## 1. Introduction 

In the international DSM-5 classifications [1], there is no specific distinction of the depressive disorder semiology between children and adults. It can affect all aspects of life, including relationships with family, friends and community and the ability to function at school or work. Several factors contribute to the prevalence of depression, which may be biological and genetic or environmental and social. The World Health Organization [2] ranked depression as the most common mental disease contributing (7.5%) to global disability, representing a worldwide public health issue that can lead to suicide. The prevalence of depressive disorder is estimated at 4.4% of the global population and anxiety disorder at 3.6%. According to the High Authority of Health (HAS) in France [3], the prevalence of depression in adolescence is estimated to be from 4% to 8% between 12 and 18 years old, while the prevalence in children is about 2.1 to 3.4%. It is known that the emergence of the COVID-19 pandemic has created an environment where many determinants of poor mental health have been exacerbated, and it has increased the prevalence of depression and anxiety [4]. However, this does not impact the current retrospective study about the trajectory of depression in high intellectual potential (HIP) children with data collected before 2017. 

Depression in children and adolescents remains a rare clinical entity (2%–5% according to the DSM-5) but the consequences on the child’s overall functioning can be serious, whether in the socialization or learning field. Difficulties of social adjustment or asynchronous development across the cognitive, emotional, social and physical domains [5,6,7,8,9,10,11,12,13,14] can also impact the outcome of depression or anxiety disorders in gifted children. Furthermore, the intellectual advancement (≥2 standard deviations from the mean IQ ≥ 120) of gifted children and adolescents, which is also called in the literature high intellectual potential (HIP ≥ 130 IQ), increases the expectations of parents and teachers about academic efficiency. A very superior intellectual quotient (IQ > 180) may be a marker of asynchronous development within academic and social environments [15].

However, the studies on prevalence of depression remain contradictory, with HIP children and teenagers having a prevalence that is neither higher [13,16,17] nor lower than non-high potential youth [16]. On the other hand, they suffered a higher rate of depression [10] compared to non-HIP children in another study. In a recent review by Kermarrec et al. [18] on the symptoms of depression in HIP children, anxiety has been mainly studied in the literature but the results are contradictory according to these studies [19,20]. Nevertheless, today we do not know what the developmental trajectory of depressive symptomatology is in these HIP children compared to HIP children without depressive symptoms. In the literature, there are various debates on the links between elements of psychological and emotional development and depression in HIP children [19]. Some emphasize links with learning disabilities [21,22,23]. However, it would be very relevant to analyze all of these factors retrospectively on the basis of anamnestic data to gain a better understanding of the factors most closely associated with the symptoms of depression in HIP children. A recent study [24] showed that the combinations of anamnestic and clinical factors allow for early and accurate identification and differentiation of psychological disorders, notably depression in mothers (postpartum depression) and children.

Generally, the methodology used for selecting high-IQ children and the sample sizes are often not adequate to provide robust results. In addition, these are mainly studies using only either questionnaires or non-validated scales or non-specific instruments without considering all aspects of mood disorders, learning disabilities and the DSM classification. Indeed, anxiety is just one of the symptoms of depression, while there is a set of other symptomatologies.

Moreover, it may result in higher level of stress and anxiety if they have an undetected learning disability because they use their capacity to compensate and so mask it with better coping skills [21,22,23,25,26]. Self-esteem is also more affected in depressive young people with a learning disability than in those without a learning disability [21,27]. Depression is reported by 15.3% of parents of HIP children according to the National Center for Assistance to High-Potential Children and Adolescents (CNAHP) in France [25], and was confirmed for only 8% of them by psychiatric judgment as depressive disorder according to the DSM-5 and ICD-10 diagnostic classifications [26]. Furthermore, according to the CNAHP’s study, learning disabilities were noted at 28.8% [25]. However, specific learning disabilities are less known or very rarely highlighted in the research about HIP people, although they can coexist with giftedness, but the difficulties remain underestimated or underdiagnosed [21,23,25,27,28,29,30,31]. Furthermore, only a few authors [21,23,25,28,32,33] warned about the heterogeneity of the IQ profile in high intellectual potential children which could be associated with neurodevelopmental disorders and lead to depression. A preliminary study showed [22] that depressive symptoms in HIP children versus non-depressive symptoms in HIP children administered with the MDI-C assessment [34] may be significantly correlated to a learning disability (e.g., 23% reading, 7% writing, 7% calculation) or a socio-cognitive disorder. 

In addition, research on HIP children has often focused on cognitive variables from the IQ scale of Wechsler. Only a small number of longitudinal retrospective studies showed that HIP children were characterized by an early maturation of the nervous system center, leading to an early psychomotor development [6,7,21,35,36,37,38,39]. However, this does not prevent learning disabilities [6,7,21,35,36,37,38,39].

Although HIP children and adolescents present advanced cognitive abilities, they should have a better aptitude for resilience [38], however, some of them will have depression [13]. Unfortunately, this state of the art in the literature does not allow us to understand, in HIP children and adolescents, the trajectories and risk factors for depression because the data collected are limited. Standardized self-report scales such as the Multiscore Depression Inventory for Children (MDI-C) [34] are specific to assess the different features of depression in children and adolescents by creating a depression severity score based on different dimensions of the depression symptomatology but often unassessed. The MDI-C is the first measure of childhood depression with items created by children, written in their own words, covering both the DSM-4 and the ICD-10 coding systems for depression following the algorithm of depression [39]. 

In the current study, we offered a holistic developmental approach taking into account the person as a whole with the different dimensions of his psychomotor development, school performance, behavior and mood, interpersonal relationships and relationship with his environment to explore the prevalence of depressive symptoms in a national sample of HIP French children seeking help from gifted organizations and to refine the trajectory of depressive symptoms. Thus, our study sample is a HIP population-based study with biased recruitment, so it does not represent the HIP population. However, it may allow us to better understand the depressive symptoms of young HIP people seeking help. We aimed to analyze the different sources of variables, collected since the neonatal period to high school, to study the risk factors of depression in the developmental trajectory of HIP children and adolescents.

## 2. Material and Methods

### 2.1. Participants 

Participants were drawn from a national retrospective survey sent to 1200 families with gifted children (IQ from preschool to high school). The sample was constituted in such a way as to be representative of the French population of high intellectual potential children related to the IQ criterion ≥ 130 (evaluated at some point in their primary or secondary schooling by a psychologist using a Wechsler scale III or IV) to belong to the “National Association for Intellectually Precocious Children” (Association Nationale Pour les Enfants Intellectuellement Précoces”, ANPEIP) in France. Thus, the children were recruited from different French regions (northeast, northwest, southeast, southwest, center including Paris, east, west). Completed questionnaires were returned anonymously for analysis.

The institutional research ethics committee of Paris Descartes University approved the study procedures performed in accordance with the Declaration of Helsinki. All the parents and the children provided written informed consent. The data collected were anonymized with a univocal numeric code assigned to each subject included in the study for analysis.

### 2.2. Design and Measures 

**Figure 1:**
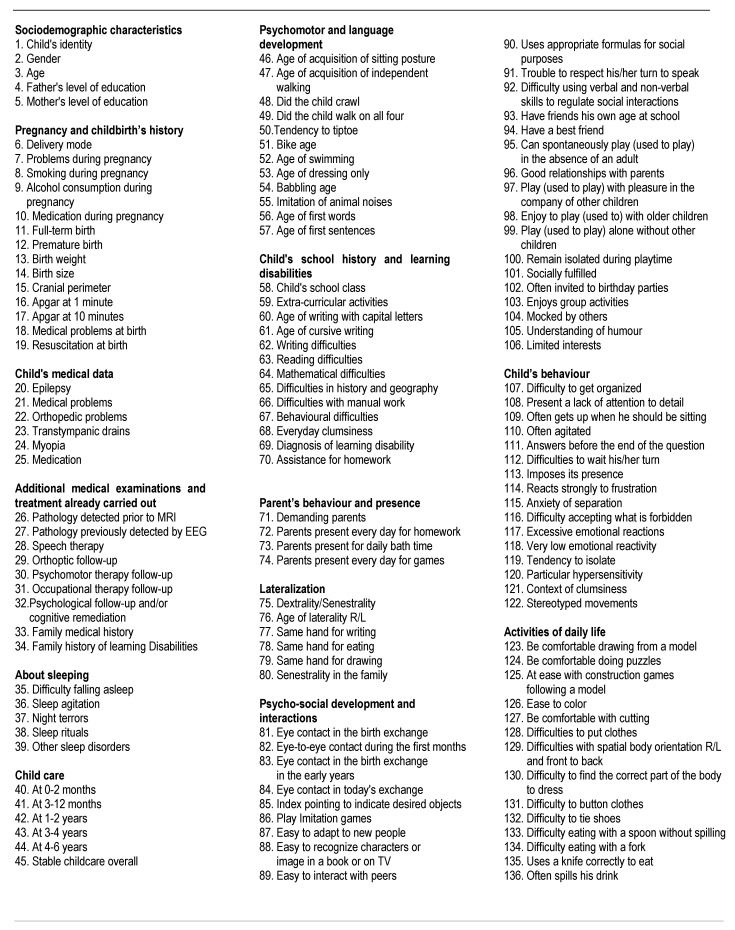
Anamnestic variables.

The children’s parents completed a self-report questionnaire as part of a larger wave of data collection on different time-points of the child development, family’s history and socio-behavioral features (136 anamnestic variables, see Figure 1): e.g., pregnancy, term and delivery mode, neonatal period, psychomotor development, health, academic school performances and schoolwork, interpersonal relationships with family and friends at school, child’s behavior and parental socio-economic status. Questions were divided into thirteen sections, and the parents had to complete the questionnaires at different time-points corresponding to childbirth, 0 to 4 months old, 4 months to 1 year old, 1 to 3 years old, 3 to 6 years old, primary school, middle school and high school. Moreover, parents had to specify whether their children present neurodevelopmental disorders based on DSM-5 criteria [1] about learning difficulty, psychomotor and/or psycho-socio-cognitive disorders, as well as therapeutic or remediation follow-up. Answers could be either quantitative (age, grades at school, for example) or dichotomic (“yes” or “no”, for questions such as “Did he/she like to draw?”).

In addition, a self-reported depression scale, the Multiscore Depression Inventory for Children (MDI-C) as a French adaptation of the original version [34], was sent to families to be administered to their HIP children. One of the key features of the MDI-C is that it allows the measurement of multiple aspects of depression. Indeed, the main objective of MDI-C is to provide truly independent measures of depression characteristics as identified in the child according to the test [34]. Children had to answer the 79 items of the MDI-C scale with either “true” or “false”. As this instrument was originally validated for children from 8 to 17 years old, we have therefore only analyzed a sample from this age range. The MDI-C has a robust psychometric property providing a screening of depressive symptoms completed by clinical evaluation, showing a high internal consistency (0.92) and test–retest reliability (subtest range of 0.77 to 0.86). This is a self-report screening standardized instrument that is completed by the child. It measures various types of symptoms associated with multicomponent domains of depression represented by eight subscale scores and 79 items of true or false statements related to how the child or adolescent generally feels: anxiety, self-esteem, sad mood, instrumental helplessness, social introversion, low energy, pessimism and defiance. It can be used as a depression severity scale through its total scale score. Raw scores are calculated by summing the number of items for each subscale as well as for the total scale and converting them into standard scores (normalized T-score, M = 50, SD = 10). Scoring profiles are separated by gender into three age groups (age 8–10; 11–13; 14–17). The MDI-C provides a measure of symptomatology levels according to the depression severity, with an MDI-C cut-off total score for each subscale and a total scale of 56 to 65 for mild to moderate depression, 66 to 75 for moderate to severe depression and above 75 for severe depression. A suicide risk indicator was considered based on specific questions included in the MDI-C. 

### 2.3. Statistical Analysis Procedure 

We used SPSS-25 software for statistical treatment. We used *p*-values at 0.05 to indicate statistical significance. To investigate developmental trajectory of depression from early childhood to high school in HIP children, we analyzed the correlations between anamnestic variables and depression in the three classes independently but also all retrospective anamnestic variables from early childhood as a single group. Thus, the Spearman’s ρ test (ρ) for non-parametric correlations was used. The Pearson’s chi-square test (χ^2^) was used to compare qualitative variables in the groups. To compare differences of quantitative means between groups (primary, middle and high school) at the MDI-C scale, we used the analysis of variance (ANOVA) test. To compare the MDI-C profile of depression in two groups (depressed and not depressed), we used the Kruskal–Wallis H because the Kolmogorov–Smirnov Z test for normality showed a *p* < 0.001.

## 3. Results

### 3.1. Sample Characteristics: Socio-Demographic and Clinical Data

**Figure 2:**
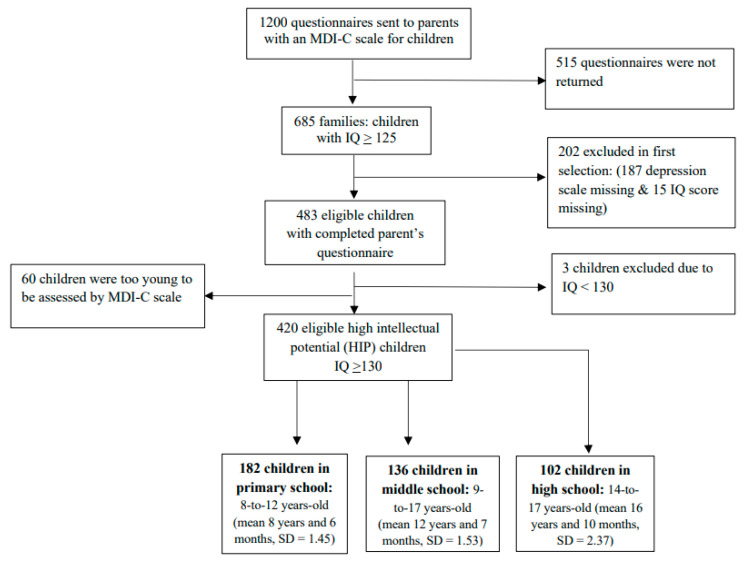
Flowchart of national and retrospective survey of included and excluded participants.

Between October 2015 and April 2017, 685 (57%) of 1200 questionnaires were returned (27% females vs. 73% males), and 420 (35%) eligible gifted children were considered as HIP (≥130) and completed the self-report questionnaire of the depression scale (MDI-C). The flow of participants according to the school level is illustrated in Figure 2.

**Figure 3:**
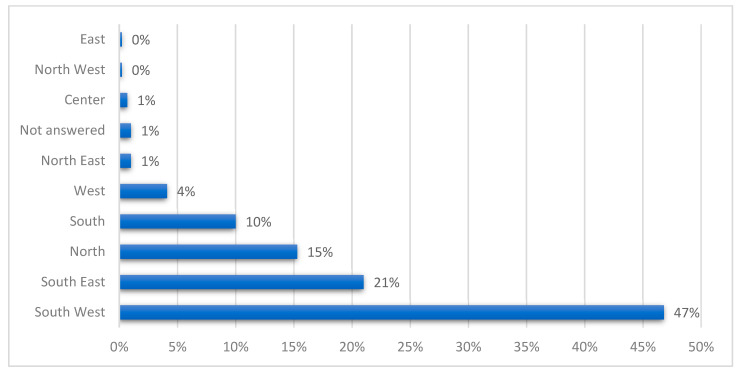
Study sample’s geographical distribution in France.

HIP children were recruited in all French regions, representing a national cohort; they were geographically distributed as illustrated in Figure 3.

**Figure 4:**
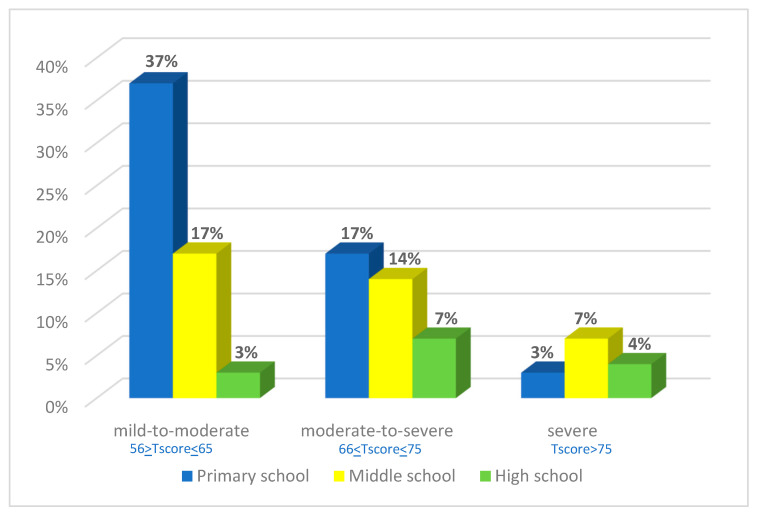
Prevalence of score levels according to the severity of depressive symptoms in the MDI-C test, in HIP children at primary school, middle school and high school.

In the 420 HIP children, we identified 205 (49%) with symptoms of depression and 215 (51%) without symptoms of depression including 57% with mild to moderate depression, 38% with moderate to severe depression and 14% with severe depression, with different degrees of severity according to the school level. We distinguished 57% depression at primary school, 38% at middle school, and 14% at high school (Figure 4). 

Considering the parental socio-professional characteristics in the study sample (Figure 1), more than half of the fathers were in the upper socio-economic category (63%) and the percentage for mothers was lower (37%). No significant difference was found between the groups with symptoms of depression and without symptoms of depression related to the socio-professional characteristics of the father and of the mother.

We noted a relevant rate of 16% of HIP children born preterm (mean gestational age 34 weeks and 7 days versus newborn at term, 39 weeks and 3 days) and 67% for complications during pregnancy. The symptoms of depression were not significantly related to prematurity (r = 0.049, *p* = 0.36) or to complications during pregnancy (r = 0.049, *p* = 0.31). Table 1 shows the details of the socio-demographic and clinical characteristics of the whole sample. Only a significant difference was shown (*p* = 0.03) between depressed and not depressed children regarding the frequency of being the second child in the family in favor of the no depression symptom group. 

### 3.2. Developmental Trajectory of Depressive Symptoms from Early Childhood through High School in HIP Children: Relationship between IQ and Depressive Symptoms and Role of Anamnestic Features

When we compared depressive symptoms (n = 205) and the lack of depressive symptoms in HIP children (n = 215), we noticed a significant difference starting at an IQ > 145 (χ^2^ (1) = 19.36, *p* < 0.0001)) with a high rate of depressive symptoms (19% vs. 10% in children with IQ < 145).

Concerning anamnestic fields, from 136 variables, only one, “learning disabilities”, was overall found significantly related to depression symptoms in different age groups of school levels, at primary (ρ = 0.45; *p* = 0.001), middle (ρ = 0.46; *p* = 0.02) and high school (ρ = 0.65; *p* = 0.001). Two other variables were significant: on the one hand, “difficulties in psychomotor skills” from childhood to primary school (ρ = 0.42; *p* = 0.003) and from childhood to middle school (ρ = 0.16; *p* = 0.02); and on the other hand, “good relationships with family as well as with peers”, which are significantly negatively correlated with depressive symptoms from childhood to primary (ρ = −0.43; *p* = 0.003) and high school (ρ = −0.31; *p* = 0.02). Furthermore, the same variables showed significant correlations with the MDI-C scale’s subfactors: “learning disabilities” was found significantly related to “low self-esteem” in all groups of school levels (ρ = 0.71; *p* = 0.001), “difficulties in psychomotor skills” with “anxiety” (ρ = 0.52; *p* = 0.002) and “low self-esteem” (ρ = 0.53; *p* = 0.01), “good relationships with family as well as with peers” with “social introversion” (ρ = −0.61; *p* = 0.001), “pessimism” (ρ = −0.39; *p* = 0.01) and “low self-esteem” (ρ = −0.57; *p* = 0.02).

### 3.3. Difference between Primary, Middle and High School Groups of HIP Children Regarding MDI-C Subtest Scale

Given that the Kolmogorov–Smirnov Z test for normality shows a *p* < 0.001, we used the Kruskal–Wallis H to compare the MDI-C subscale scores between the two groups (depressive symptoms and no depressive symptoms in HIP children). Regarding the subscale scores of the MDI-C (Table 2), they are all significantly higher in the group of HIP children with depressive symptoms (>53 T score). However, we noted a significant difference between school classes regarding a high score of symptoms: “provocation” (T mean score = 64) as a significant marker at middle school (F (1, 418) = 3.487, *p* = 0.03) and “low self-esteem” (T mean score = 77) at high school (F (1, 418) = 3.337, *p* = 0.03). 

In addition to these significantly higher scores of markers, Table 2 below shows that the depressive symptom profile assessed on MDI-C subscales in HIP children was mostly characterized both at primary and middle school by social introversion (respectively, T mean score = 63; T mean score = 63) and sad mood components (respectively, T mean score = 62; T mean score = 62), with introversion (T mean score = 62) and sad mood being replaced by pessimism (T mean score = 65) at high school.

## 4. Discussion

The current study is among the few studies that have applied a standardized assessment of depressive symptoms on a national HIP sample. In addition, data are collected on different stages of the child and family history such as the child’s social behavior since birth, with a follow-up at certain time-points of the schooling of the child from primary to high school, and also on the socio-economic characteristics of the family. 

Compared with national statistics of the general French population census from the (Institut National de la Statistique et des Études Économiques (INSEE) (National Institute of Statistics and Economical Studies)) [40], fathers and mothers in the study sample present a considerably higher frequency of managers and intellectual professions (respectively, 60% and 37%) than the general population (respectively, 20% and 15%). Only 20% of fathers and mothers belonged to the unqualified professional category vs. 43% in the general population. This also leads to a lower frequency of blue-collar workers (5% vs. 32%) than in the general population. Other professions present relatively less marked discrepancies compared to the general population, such as farmers, however, the parent population in this study has a higher social status, as shown by their professional level. Certainly, this may be because these parents are more preoccupied with ensuring a good future or career for their children or adolescents, therefore they look for help from associations if something goes wrong at home or at school. Similarly, this may be because there are more boys (76%) than girls (24%) in our sample but it is also known that HIP children and especially boys are not immune to learning disabilities, especially in non-verbal activities [7,21], and this is consistent with the DSM-5 [1], indicating a higher prevalence of neurodevelopmental disorders in boys. In our HIP study sample, it is noted that 16% of children were born preterm but we did not find a significant relationship with depression. 

In the present study, our findings show a better return of the MDI-C depression questionnaires from the south of France because it is the location of the national association of gifted children’s (ANPEIP) headquarters. 

A high rate of 49% of HIP children and adolescents in our study sample were identified as having depressive symptoms compared to the general population (4.4%) [2], with 57% mild to moderate depression in the MDI-C child questionnaire. Meta-analyses for depression and anxiety of Martin et al. [16] showed that gifted children are at lower risk for depression. However, our sample does not represent the general HIP population because it was recruited through specific associations of HIP that provide assistance. We significantly found more second children in the no depressive symptoms group, and we noted more only child in the depressive symptoms group, which may be related to the lack of daily interactions with other children at home. 

Some studies on depression [13,14,41] that compared a group of HIP children matched with a control group of non-HIP children on age, gender, socio-economic level, ethnicity and grade did not find a significant difference between the two groups. Thus, it may be not related to high cognitive abilities but it would be interesting to better understand the high prevalence of depression in our current study. 

Depressive symptoms’ severity decreases according to the school level, reaching more than half at primary school (57%), 38% at middle school and 14% at high school with more severe depression at middle school (7%) underlining the limits of resiliency among the youngest. Moreover, all the subscale scores of the MDI-C are significantly associated with depressive symptoms score, thus they do not represent just some features such as anxiety or self-esteem. 

The rate of suicide risk is very important (19%) in the whole sample studied with 7% at primary and middle school and 5% at high school, and it is known to be a consequence of high risk of depression [13,42]. However, suicidal ideation in HIP children is poorly documented. Only the study of Vaivre-Douret [6] emphasized certain comments of HIP children linked to interpersonal relationships and the understanding of the social environment (school, family, peers) such as “*Rather than die than go to school, they are mean*” (7 years old), “*Others look at me as a stranger or as if I had the plague. I feel misunderstood”* (7 years and a half), “*I want to stop suffering”, “I no longer want to make the effort to be more readable in writing”* (10 years old). The interpretation of these respective comments underlines great distress and frustration, particularly at primary and middle school before adapting to difficult circumstances (at high school). Our findings are consistent with three hypothesis points based on studies on HIP children and adolescents [6,7,14,21,25,43]: environment identification being different with advanced cognitive ability, lack of stimulations or challenges and the discrepancy between a high intellectual potential and low school performance due to learning disability impacting academic performance. 

The depressive symptoms profile assessed on the MDI-C subscales of the present study is mostly characterized both at primary and middle school by the social introversion and sad mood components, with sad mood being replaced by pessimism at high school, probably because there is a better awareness of their difficulties leading to a devaluation of the self according to psychanalytic theory and characterized by low self-esteem. However, in the literature, no studies have identified a significant difference in suicidal ideation among gifted and non-gifted youth, but it appears to depend on the methodology and on the representative sample. As suggested by Martin et al. [16], the prevalence of mental health problems would be higher among gifted children who have not been identified because they mask.

Regarding the analysis of anamnestic variables in our sample, they can be translated into either endogenous characteristics or exogenous circumstances (personality or physical features on the one hand and relationships on the other hand). We noted in the study sample an important rate of premature births (16%), not significantly related to depression, and there is no difference regarding the IQ level in HIP children born at term, consistent with a previous study [44]. 

At middle school, family relationships were not significant, while disturbing personality changes with “provocation” appeared as significant during the same period. Middle school is also a period where variables were more endogenous than exogenous (disturbing personality changes and physical motor difficulties). During high school, low “self-esteem” is a significant marker of depression as well as a certain struggle with learning disabilities and difficulties with family and peer interactions. 

The poorer the social relationships and psychomotor abilities, the higher the probability of depressive symptoms, which may lead to a suicide risk. Two important categories of variables can be noted from early childhood to high school: good relationships with family as well as with peers are significantly negatively correlated with depressive symptoms, meaning they are protective factors against depression. We can also consider the hypothesis that these two dimensions appear as an endogenous or exogenous variable regarding a disturbing personality change, leading the child to be isolated or, due to a special gift, leading them to be poorly socially adjusted due to differences. This reinforces the importance of social interactions linked to attachment, especially with parents at an early age [45,46].

Moreover, we showed a significant difference starting at an IQ > 145 with a high rate of depression (19% vs. 10% in children with IQ < 145) and having a “specific creative talent” described by parents and/or teachers. It is particularly significantly and positively correlated with depressive symptoms regardless of the degree of severity and related to their higher IQ (≥145). It may be explained, on the one hand, by a mismatch with their expectations from their environment (family, school) and they have difficulties finding peers who share their specific interests, contributing to social isolation, which in turn may lead to suicide ideation [42]. On the other hand, Karpinski et al. [47] have suggested that high IQ in HIP children would be a risk factor for psychopathological disorders, particularly depression.

Overall, positive social relationships with family and peers appear as an exogenous variable of vulnerability with a predisposition from birth in early interactions which could be intrapersonal, biological/genetic or due to exogenous disturbances in early secure attachment which could occur for any child [45,46,48,49,50]. In addition, “psychomotor skills” and “learning disabilities” appear as endogenous variables. Thus, it is not surprising that some studies [33,51,52] about HIP children showed a possible co-occurrence of neuropsychopathological disharmony with socio-cognitive disturbances which are highlighted by the heterogeneity of the IQ profile with a very high verbal score compared to the other subscales. This does not appear only in primary school or adolescence. Learning disabilities appear in our results as an important factor overall found significantly related to depressive symptoms in different age groups of school levels, and it is consistent with findings of the CNAHP’s study [25], which noted a prevalence of nearly 30% for learning disabilities in HIP children and adolescents.

Most studies [10,12,13,14,15,16] about depression in HIP children and adolescents are based on cross-sectional studies at these periods of life. Thus, the studies in the literature are contradictory, with certain authors [13,16] concluding that the prevalence of depressive symptomatology in HIP children and adolescents was not any higher or lower than in non-HIP children, while others found [10] significantly more depressive symptoms in HIP children than in their peers in a control group and they displayed more emotional disorders. However, the findings of these last studies should be analyzed with caution because they only used the Child Behavior Checklist (CBL) [53] reported by parents. 

In addition, some studies showed a link between heterogeneity of IQ profile and anxiety, self-esteem, emotional dysregulation and depression scores [26,33,51], highlighting the hypothesis of a certain psychopathological cognitive disharmony. It would also be interesting to further analyze this heterogeneity by considering other aspects of cerebral functions in the neuropsychological, neuropsychomotor, neurovisual, and psychopathological domains. Indeed, it has also been shown [7,21,27,29,30,32] that HIP children can be significantly associated with neurodevelopmental disorders that are often overlooked but have an impact on their psychoaffective behavior, particularly self-esteem and anxiety levels, as with any child. The HIP child has high cognitive abilities to spontaneously compensate or struggle with adapted strategies for their misdiagnosed disorders and, thus, may mask them a for long time [7,21,23]. Nevertheless, this compensation has an investment cost that may affect his self-esteem, and the outcome is a significant pessimistic feeling or distress at high school as highlighted in our results. If their neurodevelopmental disorders are diagnosed, our findings showed that they need special support, which is significantly correlated with psychomotor/motor skills at primary school and middle school. These include developmental coordination disorder [27], dysgraphia [23] and dyslexia [31]. These findings confirmed a preliminary study [21] which has analyzed learning disability in correlation with depressive symptoms in HPI youth versus no depressive symptoms and found a prevalence of 23% reading, 7% writing, and 7% calculation difficulties.

Furthermore, Bénony et al. [10] underlined that the loss of academic self-esteem seems to be one of the main causes of depression in HIP children and a distortion between HIP self-esteem and academic self-esteem is consistent with our findings. The authors tended to show that a problem of social non-adjustment is linked to the asynchronous expectations of the environment, ignoring or not taking into account the high cognitive aptitude of the HIP child. Indeed, psychosocial difficulties are increased by advanced cognitive ability coupled with heightened sensitivity [14,54,55,56]. However, our results allowed us to highlight a new hypothesis to better understand the risk factors of depression with a developmental trajectory origin leading to a social adjustment disorder with family and peers. A HIP child should not have all the symptomatology of depression if fortunate enough to have good coping skills or resilience [12,38].

Moreover, our results allow us to show that, linked to developmental trajectory, the clinical symptoms of depression were different between middle and high school. Provocation seems to be a significant marker symptom of depression in children at middle school, while low self-esteem is the significant marker in the HIP children at high school. At middle school, provocation behavior coincides with adolescence. It is a transition period of maturity before becoming an adult with physical and psychoaffective changes, with often maladaptive psychosocial and emotional adjustments leading to conflicts in the environment, as seen by parents. At high school, low self-esteem is the result of a developmental trajectory with many years of struggles with the environment or/and a learning disability leading to a psychoaffective disorder. Nevertheless, self-esteem is known to be more affected in learning disabilities [21,27].

Certain limitations should be considered. First, the organizations of gifted children did not cover the entire HIP population, limiting the generalizability of our findings and introducing a bias toward children with depressive symptoms. Second, we could not analyze the impact of the subscale scores of total IQ because they were often missing from the retrospective questionnaire. In addition, our study concerns HIP children with an IQ ≥ 130, and it is unclear whether our findings are specific to HIP children and it will be necessary to compare to a control group without HIP children. 

## 5. Conclusions

It is important to emphasize that depressive symptoms in HIP children could be linked to endogenous or exogenous social adjustment factors in childhood. It may also be associated with challenges in identifying and acknowledging their high abilities, as well as difficulties in developmental psychomotor skills and or psychological disorders which can worsen the intensity of depression. Studies in the literature were mainly focused on unicentric cross-sectional studies on children and/or adolescents, with bias of different inclusion criteria, and not always based on an IQ test or examining the possible associated neurodevelopmental disorders or involved recruiting children from clinical out-patients and from specific school programs for HIP children existing in the USA. 

The main strength of this current study is the provision of a database at a national level with in-depth, anamnestic data and the administration of a standardized assessment to evaluate all the dimensions of the depression symptomatology. Thus, this is an important retrospective study to assess the strength of the relationships between all these multidimensional variables. 

Finally, the current study suggests that researchers and clinicians should pay special attention to a holistic developmental approach from birth (regarding psychomotor development, mental, emotional, familial, social, and school academic performances) at all the stages of the HIP child’s development, with in-depth multidimensional clinical assessments to better understand the dynamic of causal factors by taking into account all the depressive symptoms in HIP children and comparing them with non-HIP children. 

Early identification of a risk of depressive symptoms in HIP children regarding significant anamnestic detection variables during childhood about psychomotor development and good relationships with family as well as with peers appears crucial for improving educational approaches or clinical decision making. A psychoeducational approach could be focused on parents of HIP children for a better understanding of giftedness and a well-being of the child [57,58,59]. Learning disabilities should be assessed more in HIP children because they can coexist with giftedness, but they are often underdiagnosed or underestimated, with HIP people using their ability to mask them with an investment cost and struggle. It is also important to examine the heterogeneity of the IQ subtests profile to complete assessment in the neuropsychological, neuropsychomotor, and psychopathological fields. 

It therefore seems that psychomotor therapy or psychological care for these detected children could be useful in helping them improve their physical skills, social adjustment, and self-esteem for better well-being. 

## Figures and Tables

**Table 1:** Sociodemographic and clinical characteristics of the study sample relative to the presence or not of significative depressive symptoms.

Sociodemographic and Clinical Data N = 420					
	Depressive Symptoms N = 205 (%)	Non-Depressive Symptoms N =215 (%)	χ^2^ or T-Score	Df	*p*-Value (95% CI)
**Age (months):** mean (SD)	140 (3.3)	126 (3.19)	−2.99	419	0.3 (−1.13 to −5.04)
**IQ** mean (SD)	138.76 (6.21)	138.65 (5.53)	−0.08	419	0.94 (−0.03 to 2.08)
**Gender**					
Male	156 (76.1%)	174 (74.68%)	0.98	419	0.35 (0.58 to 2.21)
Female	49 (23.9%)	41 (19.07%)	0.93		0.39 (0.17 to 2.12)
**Weight at born (kg):** mean (SD)	3.33 (0.47)	3.33 (0.51)	0.18	419	0.86 (0.13 to 2.17)
**Height at born (cm):** mean (SD)	49.92 (2.45)	49.93 (0.25)	0.15	419	0.87 (0.13 to 2.01)
**Delivery mode (n)**					
Cesarean	33	37	0.63	417	0.725 (0.45 to 2.13)
Vaginal	169	178	0.19		0.205 (0.11 to 2.22)

Df: Degree of freedom; CI: Confidence interval.

**Table 2:** Comparison of the MDI-C subscale scores between depressive symptoms and non-depressive symptoms in HIP children.

Period	MDI-C Subscale Scores	Depressive Symptoms Group (N = 205) Average Score	Non-Depressive Symptoms Group (N = 215) Average Score	Kruskal-Wallis Score	df	*p*-Value	95% CI
**Primary school**	Anxiety	60	37	100.64	240	<0.0001	98.12 to 104.12
	Self-esteem	54	27	114.19	240	<0.0001	112.34 to 116.34
	Sad mood	62	47	121.51	145.12	<0.0001	119.11 to 123.71
	Powerlessness	58	40	123.29	165.62	<0.0001	121.14 to 125.24
	Social Introversion	63	43	71.26	153.91	<0.0001	69.16 to 73.17
	Low energy	59	34	72.46	152.73	<0.0001	70.35 to 74.67
	Pessimism	58	25	109.79	142.67	<0.0001	107.34 to 111.78
	Provocation	55	41	82.43	234	<0.0001	80.45 to 85.98
**Middle school**	Anxiety	60	42	35.82	135	<0.0001	32.78 to 38.14
	Self-esteem	61	41	58.82	135	<0.0001	56.87 to 61.87
	Sad mood	62	45	54.24	128.21	<0.0001	52.76 to 56.76
	Powerlessness	61	46	65.27	135	<0.0001	63.56 to 67.83
	Social Introversion	63	41	29.62	13.72	<0.0001	27.54 to 31.65
	Low energy	58	29	31.31	134	<0.0001	29.76 to 33.45
	Pessimism	58	41	52.45	13.31	0.013	54.78 to 54.65
	Provocation	64	36	32.24	134	<0.0001	34.92 to 35.73
**High school**	Anxiety	59	38	20.57	53.84	<0.0001	18.46 to 22.34
	Self-esteem	77	35	15.67	58	<0.0001	12.35 to 17.87
	Sad mood	60	37	19.06	58	<0.0001	17.84 to 21.46
	Powerlessness	60	41	19.68	58	<0.0001	17.83 to 21.78
	Social Introversion	62	40	11.89	58	<0.0001	9.84 to 13.49
	Low energy	58	42	14.74	58	<0.0001	12.84 to 16.78
	Pessimism	65	41	5.83	58	<0.0001	3.79 to 7.94
	Provocation	56	41	16.15	54.18	<0.0001	14.74 to 18.74

T score: Results at the T student test; df: Degree of freedom; CI: Confidence interval.

## Data Availability

The data presented in this study are available on request from the corresponding author.
